# Smallholder Farmers' Livelihood Security Options amidst Climate Variability and Change in Rural Ghana

**DOI:** 10.1155/2017/1868290

**Published:** 2017-12-03

**Authors:** Sampson Yamba, Divine O. Appiah, Lawrencia Pokuaa-Siaw, Felix Asante

**Affiliations:** Department of Geography and Rural Development, Kwame Nkrumah University of Science and Technology (KNUST), Kumasi, Ghana

## Abstract

Farming as a livelihood activity in the Bosomtwe District is threatened by climate change. This paper ascertained the alternative livelihood options of smallholder farmers against climate variability and change in the Bosomtwe District. Using a cross-sectional survey, 152 smallholder farmers were sampled from 12 communities using a multistage sampling procedure. The quantitative data collected were subjected to binary logistic regression analysis, contingency tables, frequencies, and Nagelkerke tests of association, embedded in the statistical package for social sciences (SPSS) v.17. The results indicate that farmers are resorting to alternative livelihood activities that are less capital intensive and require less skill in order to secure income and household food supply. Significant determinants of farmers' alternative livelihood are age, household size, and household food supply, which were significant at *p* < .030, *p* < .019, and *p* < .012, respectively. At a 95% confidence interval (CI), these variables had lower to upper CIs for each of the EXP (*B*), respectively, at CI = 1.134–12.524, CI = 1.359–30.224, and CI = 1.781–104.561, respectively. The paper recommends that government institutes policies that will create opportunities and draw on various local/grassroots opportunities and resources to expand farmers' asset base for sustainable livelihood strategies.

## 1. Introduction

Climate change threatens the livelihood of millions in developing countries, especially the very poor because it directly affects their livelihood sources [1, 2]. Diverse cultural systems, socioeconomic conditions, and environmental exposures make household's sources of income vulnerable over time [3]. Variation in the niche of socioeconomic, climatic, and environmental factors which livelihood has adjusted to for years culminates in vulnerability trends that jeopardise the sustainability of household income and food supply. Some livelihood assets such as agricultural production knowledge and tools become redundant, influencing sustainable livelihood strategies– ways of combining and using livelihood assets [4]. Amidst such eventualities, opportunities for livelihood diversification become critical in determining community and household ability to cope with climate related stresses and shocks [5]. Climate change mitigation and adaptation efforts must therefore include livelihood diversification components to build resilience and lessen vulnerability especially among smallholder farmers. To achieve this, a broader understanding of the factors influencing and enabling livelihood decisions as well as the processes involved is needed [6].

Climate variability and change through diverse stimuli and intervening factors affect economic, social, cultural, and natural conditions of individuals and communities, altering the value and usefulness of various livelihood assets [3]. The current trend of climate change and climate variability and the resulting effect on agriculture have necessitated the adoption of alternative livelihoods opportunities among farmers in order to secure their livelihoods. Conceptually, “livelihoods” connote the means, activities, entitlements, and assets by which people make a living [7]. These are spread across social, natural, financial, human, and physical assets as outlined by DFID [4] and therefore the security of livelihoods is equally influenced by current climate change and environmental and land degradation.

The need to strengthen livelihoods has been recognised as being very necessary in climate change and climate variability mitigation and adaptation efforts [5]. Developing adaptive capacity to minimize the damage to livelihoods from climate change is to this end a necessary strategy to complement climate change mitigation efforts. In the Bosomtwe District of the Ashanti region of Ghana, smallholder agriculture continues to be the main economic activity that sustains the livelihoods of most farmers' households. In the midst of climate variability and climate change, farmers' households and indeed the district's food security seem threatened. Until now, little scientific studies have ascertained the level of this insecurity among smallholder farmers in the district. It is a truism that farmers over the years have alternatively engaged in some form of livelihood supplements; however, it remains unclear as to the attributions of the recent intensification of the search for alternative livelihood activities among smallholder farmers.

In parts of Eastern and Southern Africa, climate change has negatively affected agriculture, water sources and quality, biodiversity, health, and ecosystems which are key components of local livelihood assets [8]. The rate of change has marginalized already vulnerable livelihoods, made those that could have adapted more slowly less adaptive, and handicapped new livelihood opportunities in the near term [9]. The impact of climate change on livelihoods is anticipated to be greater in Africa compared with other parts of the world [10]. In Africa, some livelihood alternatives that farmers resort to include seasonal migration of livestock keepers and distribution of livestock herds in different places; rainwater harvesting; doing casual labour to be able to get food and other household needs; selling of livestock; engagement in small businesses including shops, local restaurants, and kiosks [11]. Vulnerability of an individual depends on his/her assets base and the choice pattern and use of these assets available. With limited livelihood assets, the response of vulnerable individuals and communities could be unsustainable or even maladaptive. Inefficient institutional policies and processes could amplify shocks and stresses at the local level [5]. This restricts livelihood strategies and, correspondently, livelihood outcomes.

Reduction in rainfall, variation in rainfall patterns, droughts, and high temperatures are some evidence of climate change in Ghana. These have affected the livelihoods assets of communities exposing them to hunger and poverty [12]. Floods and bushfires caused by high temperatures destroy farmlands, biodiversity, and wild life which are the basic natural capital that rural people depend on for their livelihood [13]

Diversification, encompassing migration, nonfarm work, and social support networks, in addition to livestock production, according to Roncoli et al. [14], has served to moderate farming households from the adverse effect of climate variability on household income supply in Burkina Faso. Hunting and gathering of wild fruits, charcoal production, and chain saw operations are important coping strategies and a means of building assets that have become common in Ghana [15, 16]. Armah et al. [17] include petty trading (sale of foodstuff, spices, dye clothing, and other basic household needs and equipment at community levels on table tops or small shops in communities or carried from community to community on head pans), craftsmanship, production of charcoal, and selling of firewood and emphasise that, in Ghana, people's livelihood depends on farming and other off-farm income generation activities.

Most farmers usually migrate to more vibrant and economically productive areas to sell their labour. To this Nassef et al. [18] assert that many pastoralists move to urban settlements in search of alternative livelihoods. Demeke and Zeller [19] explain that when the rains are poor, farmers commit more labour resources to less risky alternative livelihood activities. Hence, sale of labour to off-farm livelihood activities lessens the effect of climate variability on household income and food supply. Eshetu et al. [20] in their study on income diversification in Ethiopia report that more than 40% increase in household income came from sale wage labour.

Migration, sale of livestock and fowls, and menial jobs such as weeding the farms of others who are able to build some resilience (among men) or fetching water (among women) for income are some of the off-farm adaptation practices outlined by Gyampoh et al. [21] in selected communities in the High Forest, Coastal Savannah, and Guinea Savannah ecological zones in Ghana. Also, the collection and sale of shea nuts* (Vitellaria paradoxa)*, dawadawa* (Parkia clappertoniana)*, fuel wood, and wild fruits have become major livelihood options, especially in the lean season in savanna regions in Africa while small-scale mining, harvest of timber for logging and crafts, and fruit gathering are some forest livelihood activities that have been intensifying over the years [12, 22]. In 2007, the three northern regions of Ghana experienced flooding that destroyed houses, displaced families, and destroyed farmlands eroding natural, social, and physical assets. Households lost their livelihoods and the resources to engage in alternative livelihood activities were scarce [13]. Amidst such dire situations, the collection of shea nuts* (Vitellaria paradoxa) *and dawadawa served to provide a source of income in the short term.

While most farmers have sought alternative livelihood options, there have been some exceptions to this trend among African farmers. According to Eshetu et al. [20], involvements in alternative income generating activities besides agriculture had not been prioritised in some parts of Ethiopia. This though not clearly outlined could be due to some form of security that complements such needs. The United States Agriculture Department [23] purports that the relevance of alternative livelihoods differs from farmer to farmer, reducing as farm output increases. Livelihoods analysis focuses on the structures and systems that determine people's asset base and the resulting livelihood strategies and outcomes [4]. These strategies and outcomes depend on the stress and shock encountered and the vulnerability context [24]. It is the human (individual and community), environmental, and ecologically differentiated vulnerability and risk arising from exposure to current and future climate change and variability that have necessitated global, regional, and national action on adaptation and mitigation. Such differences in the West African subregion as coastal, forest, savannah, and Sahel regions should form the basis for appropriate adaptation interventions in the subregion [25] and hence sustainability of livelihoods.

Ecosystems serve as a stock of diverse resources providing food, water, raw materials, medicine, and other essential ecosystem services that support livelihoods directly and indirectly [8]. In Ghana, communities on the fringes of forests utilize timber and nontimber forest products for a wide array of livelihood engagements. Anything that negatively impacts these forests equally threatens the livelihood of these forest dependent communities [12]. Engagements such as illegal mining and illegal logging however degrade the environment and as such undermine the sustainability of forest dependent livelihoods [26]. Agriculture and extraction of forest resources are the traditional sources of income for households within the* Offin* river basin in Ghana. These have however decreased considerably in recent times. Although most of the off-farm livelihoods of communities in the river basin are derived from its forest, their activities have conversely degraded it, decreasing the carbon stock of the forest [12]. This underpins the need for integrating community needs and participation in policy formulation and implementation. Inclusive and meaningful participation of all community groups, particularly the most vulnerable, is needed as they may be the very ones to spearhead unsustainable resource use at the community level.

Agriculture is the primary occupation in the Bosomtwe District which employs 62.6% of the district's labour force with crop farming and fishing, respectively, constituting 57.4% and 5.3%. In the rural communities, more than half (60%) of households are agricultural households implying that they depend on agriculture for household income and food supply. However, most rural households (97.6%) are involved in crop farming which essentially contributes to household food and income supply [27]. However, this role of agriculture as a primary livelihood activity has been challenged in recent times. According to the Bosomtwe District Assembly Profile [27], climate change is increasingly having a negative impact on agriculture through changes in the onset of rains, shifts in peak rainfall months in the major and minor agricultural seasons, droughts, and variations in temperature and flooding with direct impact on crops and rendering traditional agricultural production practices ineffective. The presence of Lake Bosomtwe as a tourist site, water and forest resources, and fast growing periurban centres presents opportunities to engage in varied alternative livelihood activities to safeguard household income and food supply. However, this has not received much research attention. This is the basis for making original contribution to literature in general and specifically within the Ghanaian context. The aim of this paper is therefore to ascertain the extent of climate variability and change-driven alternative livelihood security dynamics among smallholder farmers in the Bosomtwe District of the Ashanti region of Ghana.

## 2. Materials and Methods

### 2.1. Profile of the Study Area

**Figure 1:**
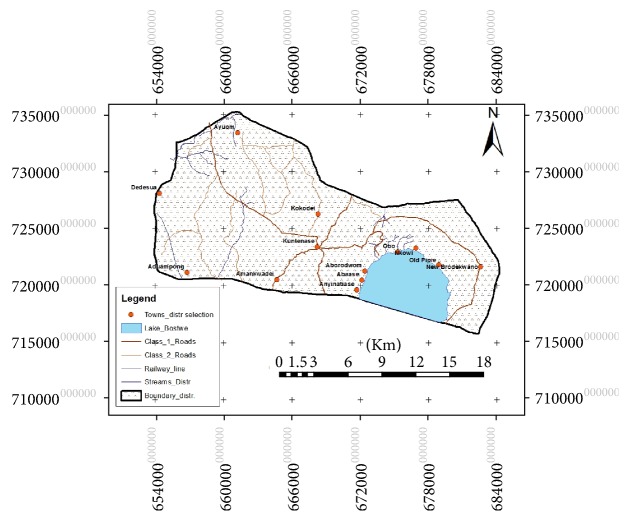
Map of the Bosomtwe District in Ghana. Source: author's construct (2015).

The study was conducted in the Bosomtwe District which lies within latitude 6°28′N–latitude 6°40′N and longitudes 1°20′W–longitude 1°37′W, at the centre of Ashanti region with Kuntenase as the district capital. The district covers an area of 330 km^2^ (Figure 1).* Atwima Nwabiagya* and Kumasi Metropolis are to its north while* Ejisu-Juaben* Municipal is to the east. The south is bounded by Amansie west and east districts [27].

The largest natural Crater Lake in West Africa—Lake Bosomtwe—is found in this district which is a major source of livelihood for surrounding communities such as fishing and other related trades [28, 29]. The dendritic pattern of drainage is characteristic of rivers and streams in the Bosomtwe District with streams flowing from the surrounding highlands into the lake. Some of the rivers in the district include* Supan*,* Butu, Oda, Adanbanwe, *and* Siso* [28].

The district is within the moist semideciduous forest ecological zone with a major and a minor rainfall regime. The major rainfall regimes is from March to July while the minor is from September to November. The ecological zone has mean annual rainfall and mean monthly temperature of about 1,900 mm and 36°C, respectively. Relative humidity also ranges between 60% and 85% [28]. The district has different tree species that have high economic value including* Mahogany (Khaya ivorensis), Onyina (Ceiba pentandra), Wawa (Triplochiton scleroxylon), Asanfena (Aningeria *spp*.) and Denya (Cylicodiscus gabunensis)*. However, due to extensive farming activities in the area, the original vegetation has been degraded to a mosaic of secondary forest, thicket, and regrowth with abandoned farms of food crops and vegetables [27].

In certain parts of the district, however, the original forest cover has been turned into secondary forest and grassland through indiscriminate exploitation of timber and inappropriate farming practices such as the slash and burn system and illegal gold mining activities [27]. The population of the district according to the Ghana Statistical Service Census is 93,910 with an urban to rural population ratio of approximately 1 : 2 [27]. Proximity of the district to the Kumasi Metropolis is greatly encouraging the growth of settlement in the district. Moreover, the district's tourism potential has drawn a lot of investments in infrastructure development and other socioeconomic activities into the district [28].

### 2.2. Sampling Design, Instruments, and Data Analysis

This study was one of the objectives of a major study on smallholder farmers' mitigation of and adaptation to climate change and climate variability in the Bosomtwe District. The specific objective was to analyze alternative livelihood activities of smallholder farmers as a means of coping with climate change in the Bosomtwe District. Key areas of concern included alternative income generating activities, length of years of being engaged in these activities, effects of climate variability on agricultural livelihood, and possibility of switching to alternative income generating activities as primary occupation. There are sixty-six (66) communities in the Bosomtwe District. These were purposively clustered into communities around Lake Bosomtwe (52 communities) and those farther away from the lake (14 communities). The lake has an outer ridge with an elevation of 50 m to 80 m which is the only unique topographical feature in the district that distinguishes communities around the lake from other communities in the district. Six communities were therefore selected from each cluster using the lottery method without replacement to aid a comparative analysis of the various ramifications of alternative livelihoods in these clusters in response to climate change relative to natural resources and different topographical terrains. A total of 152 smallholder farmers were sampled from these communities and the respective sample for each community was allocated by quota using the proportionate sampling. These are smallholder farmers who basically produce crops in order to subsist and have farm sizes ranging within 1 acre to 5 acres. They use basic/rudimentary farm tools, inputs, and technology and basically rely on household members as farm labour. The respondents for the study were sampled using simple random sampling technique. A semistructured partially precoded questionnaire was used to gather quantitative data for the study. The terrain of farmlands and the peculiar physical and built environments of the communities were also observed in relation to livelihood activities and possibilities.

The quantitative data gathered were subjected to multiple regression analysis, contingency tables and frequencies and Chi-square tests of association, and multivariate distribution of variables, respectively, embedded in the Statistical Product and Service Solutions (SPSS) v.17 Windows application. The results were displayed in tables, charts, and graphs. The diagrams generated in the SPSS were exported to Excel for editing for better visual presentation.

Farmers' disposition to increasingly engage in alternative livelihoods was ascertained. The likelihood of a farmer devoting more attention and resources to an alternative livelihood activity other than farming was analyzed using binary logistic regression modelling. Using the odd ratios for the likelihood of occupation modification, this modelling was to determine whether farmers are more likely to devote more resources and time to alternative livelihood activities besides farming.

Hence, respondents were to indicate 1 = yes, the current crop yield encourages them to consider other alternative livelihoods, and 0 = no, the current crop yield does not encourage them to consider other alternative livelihoods. The step-wise logistic regression was run in the SPSS software. The model without predictive variables was taken as the null hypothesis, which therefore states that there is no significant difference between the dummies (yes and no)—the response variables. In other words, the model would better predict the outcome without the inclusion of the independent variables, while the alternative hypothesis states that the model would not predict better without the independent variables. This means there is significant difference in the dummies in the response variables.

In the first step of the model (block 0), the model without the inclusion of the predictive variables, measured at *y* = the constant, indicated the overall percentage correctness of prediction was 92.7% with a probability value of significance at *p* < .000, with an *B*(EXP) = 0.079. By adding the predictive variables to the alternative model (block 1) equation, the overall percentage correctness of the prediction was 93.3% slightly better than the null model at a significant value of *p* < .000, with a Chi-square value *χ*^*2*^ = 30.119 at 5 degrees of freedom. The* Nagelkerke R*^2^, which is a pseudo coefficient of determination, indicates that the model can offer only 44.6% explanation of the variation in the dependent variable.

There were eight independent variables (noncategorized), entered into the alternative hypothesis equation. However three of them were significant in predicting the farmers' disposition to consider alternative livelihood activities other than farming. These independent variables were as follows: the age of the farmer, size of household, and security of household food supply. Age was significant in predicting farmers' likelihood of considering alternative livelihood activities because most of the farmers who were young could not envisage any improvement in the current trend of low crop yield in the near future. Another predictive variable is the size of household. Most farmers were of household made up of six or more members. There was hence the need for those who were of age to contribute towards the upkeep of these households. This requires a source of income and hence sustainable livelihood. The last significant predicting factor was security of households' food supply. The insecurity of household food supply due to crop failure or poor crop yield consequently necessitated the adoption of alternative livelihood activities to ensure a constant supply of household food.

Qualitative data was gathered from key informants in appropriate institutions through interviews using an interview schedule to gain in-depth knowledge on alternative livelihood activities in the district. The key informants were the District Extension Officer, District Crop Research Officer, and the Director for Women in Agricultural Development (WiAD). These were purposefully selected because they have acquired thorough knowledge about agriculture and other livelihood activities among farmers in the district. This was conducted in English. Their responses were grouped under various themes and integrated in the discussions under the various thematic treatments of the sections of the paper.

## 3. Results and Discussion

### 3.1. Sociodemographic Characteristics of Respondents

Analysis of the sociodemographic characteristics of the smallholder farmers revealed that alternative livelihood engagement plays a critical role in the adaptation process of smallholder farmers. Majority of the respondents (46%) were of the age group 46–55 years. The next two successive categories were the 36–45 years and 56–65 years who represented 33% and 21%, respectively. A total of 79% of respondents were less than 56 years implying they were young and energetic and if resourced could engage in active economic activities. Most of the respondents (82%) were married and therefore needed to cater for their households through diverse economic activities. Hence, engaging in sustainable livelihood activities was imperative for them. Also, 51% of respondents were household heads while 46% were spouses of household heads and the remaining 3% were other relations.

The communal nature of the societies is such that information is not restricted to household heads. Frequent migration among males, especially in the lean and dry season, coupled with the fact that much of household spending is done usually by the women makes them adequately informed on household spending patterns. Okonya et al. (2013) found that female-headed households in Niger were less likely to respond to climate change than male-headed households. This was probably because, in the traditional African setting, it is a man's duty to ensure that household food supply is secured and women in most cases are expected to play complementary roles. This was however not the case in the Bosomtwe District as both male and female heads made effort to adapt.

The study revealed that 63% of the respondents had formal educational level below senior high school while only 11% were educated up to the senior high school and only one percent had tertiary education. Also, 25% had no formal education as is evident. A low level of education was generally observed among farmers and this could affect their ability to diversify into livelihoods in the service and commerce sectors as was found by Wamsler et al. (2012) in El Salvador and Brazil. Majority of the farmers (90%) had household size of 1–10 members implying there were many mouths to feed and with climate change posing a major threat to agriculture as a primary occupation; securing sustainable alternative source of income was not optional. Apata (2011) likewise found in Nigeria that a larger family size increased the need to diversify income sources.

### 3.2. Disposition of Farmers to Consider Changing Occupation from Farming

In analyzing farmers' disposition to increasingly engage in alternative livelihoods, it was found that age of the farmers, size of household, and security of household food supply were significant in the likelihood of predicting the outcome of response variable. These were significant at *p* < .030, *p* < .019, and *p* < .012, respectively. At a 95% confidence interval (CI), these variables had lower to upper CIs for each of the EXP(*B*), respectively, at CI = 1.134–12.524, CI = 1.359–30.224, and CI = 1.781–104.561, respectively. Furthermore, six out of the eight independent variables had (EXP)*B* > 1 of likelihood to predict the outcome of the dependent variable of engaging in other alternative livelihood activities when those independent variables were used on the response variable at *B*(EXP) = 1.054–13.644 times (Table 1). This implies the possibility of farmers engaging in livelihood activities besides farming can be predicted by the independent variables at thirteen odd likelihoods, thus thirteen times likely to happen.

Now we substitute the variables in

(1) p=e1.327Ag+1.858Sz+2.613Hfs−49.0681+e1.327Ag+1.858Sz+2.613Hfs−49.068.

The *B*(EXP) values expressed in the equation indicate that the odd likelihood of prediction is more than thirteen times the likelihood for the farmers to continuously engage in alternative livelihood activities besides farming if their crop output remained as it is currently, due to climate variability and change.

### 3.3. Smallholder Farmers' Alternative Livelihood Activities

**Figure 2:**
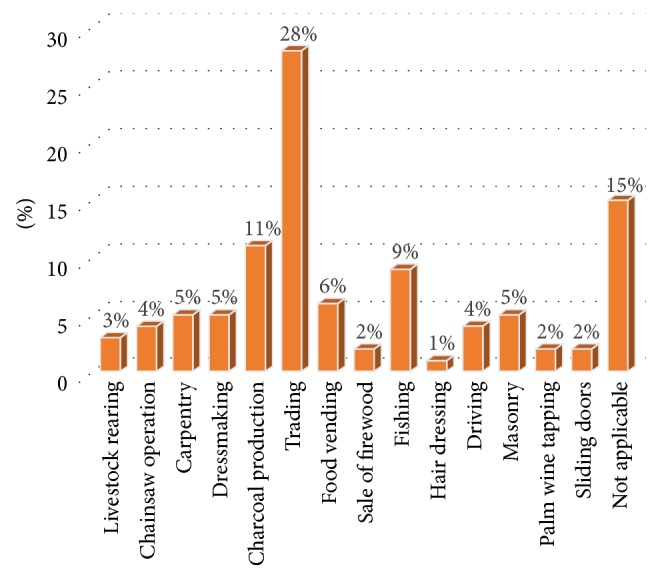
Alternative income generating activities.

Alternative livelihood activities by smallholder farmers in the Bosomtwe District are varied as can be seen in Figure 2. These are basically aimed at diversifying the income sources of smallholder farmers, hence making them less vulnerable to the impact of climate variability.

The choice to engage in an alternative livelihood activity for most of the smallholder farmers in the Bosomtwe District is influenced by current crop yield trends as more than half (61%) of respondents have resorted to alternative livelihood activities due to crop failure and low yield. The next highest figure of 24% also engaged in these livelihood activities without any climate related reason while 15% of respondents do not engage in any livelihood activity. These are farmers who depend solely on agriculture as a livelihood and rely on remittances and other forms of social support. The district coordinator for Women in Agricultural Development (WiAD) which serves to help farmers, particularly women, to engage in alternative income generating activities explained that off-farm income generating activities in the district have increased in the last fifteen years and have been skewed towards trading, logging, and small-scale mining. The latter however did not come up at all. This could be due to the fact that those who engaged in these activities may not have acquired the legal rights to undertake such activities and hence do not feel safe giving out such information. However, interactions with the key informant revealed that it is one of the major alternative engagements that has emerged in recent years. She adds that “in view of weather changes farmers have increasingly engaged in other livelihood activities, particularly petty trading, processing and sale of gari (a popular local food made from cassava) and soap making.”

Simbarashe [30] points out that farming in Bikita in Zimbabwe has been adversely affected by climate variability resulting in failure of crops and low crop yields. Farmers have thereby diversified into alternative livelihood activities such as firewood trade and brick moulding. Smallholder farmers in the Bosomtwe District point out being limited in resources to engage in these activities. One would expect trading to be prevalent in communities located in proximity to the lake due to its tourist attraction. In contrast the seasonal patronage or use has not sustained economic activities in this regard. Also fast growing periurban settlements in communities farther away provides a sustained market for petty trading in communities farther away.

The study revealed that most smallholder farmers in the district are into petty trading representing 28% of respondents. This, as observed in the communities, ranged from the sale of cooked food, foodstuff, small household appliances and their accessories, and clothes among many others on table tops and stores while others carried these from one community to another on head pans. Its predominance was because it requires relatively less capital to commence as was also asserted by Mitullah [31]. Also 15% of respondents did not engage in any alternative livelihood activity. They relied on remittances from family members in urban centres and social support systems. The next highest alternative livelihood activity is charcoal production accounting for 11% of alternative activities. Abundance of trees suitable for charcoal production was found to be the prime driving force for the practice. Farmers may be considering making the most of the current situation while seeking to secure other sources of income as the sale of foodstuff can earn a farmer enough capital to engage in petit trading. This also explains why charcoal production is prevalent since there is an abundance of trees in forests and secondary growth in the district. In tandem with this, charcoal production in the northern region was graded as the second major occupation to agriculture and also placed second with respect to income generation [32]. Agyeman and Lurumuah [33] also confirm commercial charcoal production to be a major source of livelihood in the northern parts of Ghana.

Charcoal production as the second highest alternative livelihood activity is a maladaptive practice which serves to amplify climate variability. Local climatic conditions can be exacerbated through the release of carbon sequestered in trees into the atmosphere. The 15% of respondents who did not engage in any alternative livelihood activity corresponds to findings by Shewmake [34], who analyzed the vulnerability and impact of climate change in South Africa's Limpopo River Basin. In his study he observed that majority of households did nothing in times of droughts. Smallholder farmers in the Bosomtwe District rather resorted to short term coping strategies as borrowing food and money from neighbours and relatives, migrating, and changing eating patterns to get by the situation.

### 3.4. Length of Years of Engagement in These Activities

**Figure 3:**
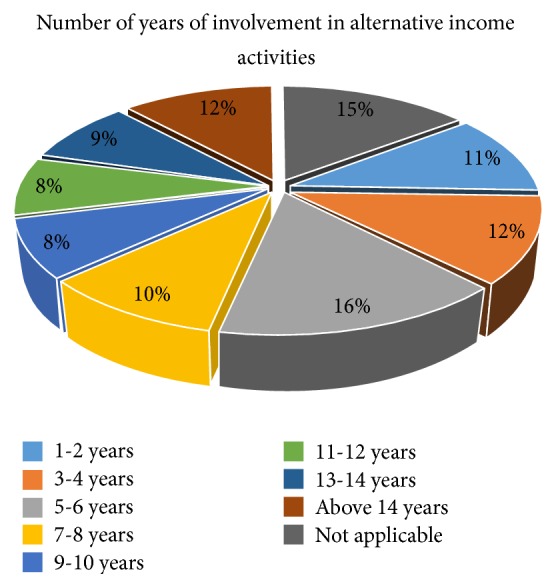
Number of years of involvement in alternative income activity.

The number of years in which smallholder farmers have engaged in these alternative livelihood activities was categorize into eight groups as follows. The percentage of smallholder farmers who have been involved in alternative livelihood activities at least within the last 15 years in the Bosomtwe District as the sum of the years ranges from 1 to 14 years is 73% (Figure 3).

Probing further to ascertain whether the shift from agriculture to other livelihood activities is likely to persist among smallholder farmers, they were asked if they were still considering other sources of livelihood and the place of agriculture if such livelihood avenues open up. Ninety-three percent (93%) of respondent were still considering other sources of livelihood activities other than that which they already practice alongside agriculture while only seven percent were content with their current economic activities—agriculture and/or current alternative livelihood activity. Of those still considering alternative livelihood activities, 60% said they would keep agriculture as their primary occupation and 33% intended to rather keep agriculture as a secondary occupation if only the alternative livelihood activity has more prospects than agriculture. For the remaining seven percent, this was not applicable. Barrett et al. [35] espouse that as output from farms decline, farm household in order to reduce risk and vulnerability naturally diversifies into off-farm activities. The main reason for diversification in Nigeria as observed by Abimbola and Oluwakemi [36] was limited agricultural income.

The role of these alternative livelihood activities is even more crucial in the district as the level of remittances is very low among smallholder farmers. Only 23% of respondents receive remittances and 77% do not. Most of those who receive remittances only received it on average twice in a year. This contextualizes the relevance of these alternative livelihood activities among smallholder farmers in the Bosomtwe District as being a very critical adaptation strategy. This is also indicative of the fact that given an exposure to a stimulus such as climate change that makes a source of livelihood vulnerable, smallholder farmers naturally seek security in other livelihood activities that are not directly affected by such a stimulus. This could be due to the fact that their current exposure to the eminent threat makes them repulsive to anything that has direct dependence on it. Hence, smallholder farmers generally looked in the direction of other sources of livelihood other than that which is directly affected by climate. This concurs with the findings of Roncoli et al. [14] who observed in their study of farmers in Central Plateau in Burkina Faso that nonfarm activities were becoming important and already formed the main source of income for households.

### 3.5. Effects of Climate Variability on Agricultural Livelihood

A cross tabulation of farmers' perceived rainfall trend in the last 15 years and considerations of practicing agriculture as a primary or secondary occupation revealed the possibility of farmers switching to alternative livelihood activities as their main economic activity due to the predictability of rainfall pattern or otherwise if better livelihood opportunities emerge (Table 2). Farmers' perceptions of rainfall trends in recent times was in agreement with meteorological data which revealed that there had been shifts in the onset of rains, considerable variations in the peak rainfall months in the major and minor season, and abnormalities in the traditional bimodal rainfall regime demonstrating unimodal rainfall regimes in some years.

Only 9% of respondents who intend to keep agriculture as their primary livelihood activity said the rainfall pattern has been consistent and predictable while 91% said it has been inconsistent and unpredictable. This conforms to the findings by Ontoyin and Agyemang [37], whose study in northern Ghana indicates that smallholder farmers are increasingly resorting to alternative livelihoods. Those intending to keep agriculture as a primary livelihood activity gave reasons as having comfort in their native communities and lack of finances to venture into the seemingly capital intensive alternative livelihood activities.

Of those who intend to switch to alternative livelihood activity as their primary livelihood activity, only six percent perceived the rainfall pattern to be consistent and predictable while 94% perceived it to be inconsistent and unpredictable. This indicates that livelihood activities amidst climate variability are threatened, especially those that are climatic dependent like smallholder farming. This gradual trend is stimulated by the fact that annual and seasonal weather patterns have increasingly become very unpredictable. Hence income from agriculture has equally become unstable. Engaging in an alternative livelihood activity is therefore a critical adaption measure which in the short to medium term helps cushion smallholder farmers from the economic implication of the vagaries of the climate. This is because rural nonfarm activities provide alternative economic livelihoods for the rural poor who have limited assets [38].

A cross tabulation of the location of communities (either located near Lake Bosomtwe or located farther away) and alternative activities revealed that some alternative livelihood activities are dominant either in communities close to the lake or farther away while others are evenly spread in the district. Trading which was the most prevalent alternative livelihood activity is more common in communities farther away (16%) rather than those near the lake (12%). This trend could be due to the fact that communities close to the lake tend to have fishing as a dominant alternative livelihood activity which is absent in communities farther away. Engaging in trading does not require any special training or skill and requires less capital [31], hence its high level among smallholder farmers in the district.

However, communities close to the lake found fishing to be a more attractive alternative livelihood activity. This is due to the generally high demand for fish in households in the district and beyond and also the ease of access to the lake due to their proximate locations. Hence fishing seemed less burdensome and more attractive compared with trading. The decline in their engagement in trading is buffered by fishing activities. Fishing and sale of firewood as alternative livelihood activities recorded higher response in communities near the lake (two percent for fishing and nine percent for fishing) as compared to those farther away (recorded as zero for these livelihood activities). Sale of fire wood is high because it serves to provide fuel wood for food vending activities which also recorded 4.6% in communities close to the lake compared with one percent in communities farther away from the lake. Carpentry and livestock rearing were widespread among the communities which are farther away (three percent each) rather than in those closer to the Lake Bosomtwe (zero percent). That of carpentry corresponded to chainsaw activity as a livelihood activity which is equally high in communities farther away from than those near (chainsaw: three percent and two percent for communities farther away from and those near) Lake Bosomtwe, respectively. This among other factors can be attributed to the topography of the land/forest around the lake which is undulating and hence difficult to access compared to that of those farther away with a gentler gradient. This seems to have fueled carpentry as an alternative livelihood activity in the communities farther away from the lake due to easy access to timber/wood. The other alternative livelihood activities were evenly distributed in the district regardless of whether the communities were close to the lake or farther away.

These alternative livelihood activities are usually done concurrently with agricultural activities and only intensified in the minor rainy season (September-October) and dry season and when crops do not do well in the major season. Similarly Roncoli et al. [14] examining farmers' response to drought conditions in Burkina Faso found that off-farm alternative livelihood activities were predominant in the dry season when agriculture produce is being sold and hence people have more money and time on their hand.

## 4. Conclusion and Recommendation

The study sought to explore the alternative livelihoods of smallholder farmers in the face of climate variability and climate change in the Bosomtwe District in Ghana. It has espoused the nexus between climate change and smallholder farmers' livelihood activities as adaptation strategies in the Bosomtwe District which are varied and complementary. Also smallholder farmer's involvement in alternative livelihood income activities has been high within the last 15 years with the prime objective of securing household income supply. The study also found that farmers generally engaged in activities that are interdependent. These were based on the various opportunities and resources available to them creating a form of specialized microeconomic livelihood system. Resource availability and use and the extent to which government policies and interventions actually reach the grassroots vary, resulting in varied local response to stimulus such as climate change. This implies that anything that affects one livelihood will have reverberations on another. This also implies that policies at advancing key livelihoods would go a long way to improve others within the district. It is therefore recommended that the Bosomtwe District assembly should expand its collaboration with NGOs and other private entities to provide training, skill development, and capital to strengthen local alternative livelihood activities and create opportunities to expand the livelihood options available. Charcoal production and illegal chainsaw operations are also prominent and have been sustained by the abundance of trees species for the practices. This is a maladaptive practice that amplifies global climate change by releasing carbon dioxide sequestered by trees into the atmosphere. Hence, the government through the Forestry Commissions must initiate and implement policies that regulate access to and utilization of timber and nontimber forest resources sustainably.

## Figures and Tables

**Table 1:** Logistic regression table of variables in the equation.

Predictive variables	*B*	S.E.	Wald	df	Sig.	Exp(*B*)	95.0% C.I. for EXP(*B*)	
							Lower	Upper
Age of respondent (*A*_*g*_)	1.327	.613	4.685	1	.030	3.768	1.134	12.524
Type of household	17.435	1.504*E*4	.000	1	.999	3.733*E*7	.000	—
Marital status of respondent	.708	.456	2.413	1	.120	2.030	.831	4.958
Size of household (*S*_*z*_)	1.858	.791	5.510	1	.019	6.408	1.359	30.224
Location of community	−1.783	.998	3.191	1	.074	.168	.024	1.189
Increased expenditure on agricultural inputs	.053	1.070	.002	1	.961	1.054	.129	8.582
Reduced income from agriculture	.623	1.037	.361	1	.548	1.865	.244	14.243
Insecure household food supply (*H*_fs_)	2.613	1.039	6.326	1	.012	13.644	1.781	104.561
Constant	−49.068	3.009*E*4	.000	1	.999	.000		

*Note*. Significant at *α*_0.05_.

**Table 2:** Cross tabulation showing farmers switching to alternative livelihood activities as their main economic activity based on the consistency and predictability of rainfall.

		Perceived rainfall pattern in the last 15 yrs		*Total*
		Consistent and predictable	Inconsistent and not predictable	
Consideration of agriculture as primary or secondary activity with respect to alt. livelihood activity	Primary occupation	8 (9%)	83 (91%)	91 (100%)
	Secondary occupation	3 (6%)	47 (94%)	50 (100%)
	Not applicable	2 (19%)	9 (82%)	11 (100%)
	*Total*	13 (100%)	139 (100%)	152 (100%)

## Additional Points

*Limitations of the Study*. Although the objectives of the study were achieved, there were inevitable limitations. The research was required as part of an academic exercise to be done within a specified time frame. This required that the study be narrowed down to specific areas in order to be feasible within the stipulated time frame. This coupled with the financial challenge underpinned the need to restrict the study to smallholder farmers in 12 out of 64 communities. Moreover, components of vulnerability of the farmers could have been included to aid a more comprehensive evaluation of agriculture vis-à-vis the various alternative livelihood activities. The few years of research and academic activity have also handicapped the scope and depth of discussion on the various thematic areas considered.
